# Supplementary material for: Role of Knee and Ankle Extensors’ Muscle-Tendon Properties in Dynamic Balance Recovery from a Simulated Slip
Source: Sensors (Basel). 2022 May 3;22(9):3483. doi: 10.3390/s22093483 (PMC9104373; doi:10.3390/s22093483)
Supplement: Supplementary file 1 [file sensors-22-03483-s001.zip › sensors-1685584-supplementary.pdf]

Supplementary material: Role of knee and ankle extensors' muscle-tendon properties in dynamic balance recovery from a simulated slip.

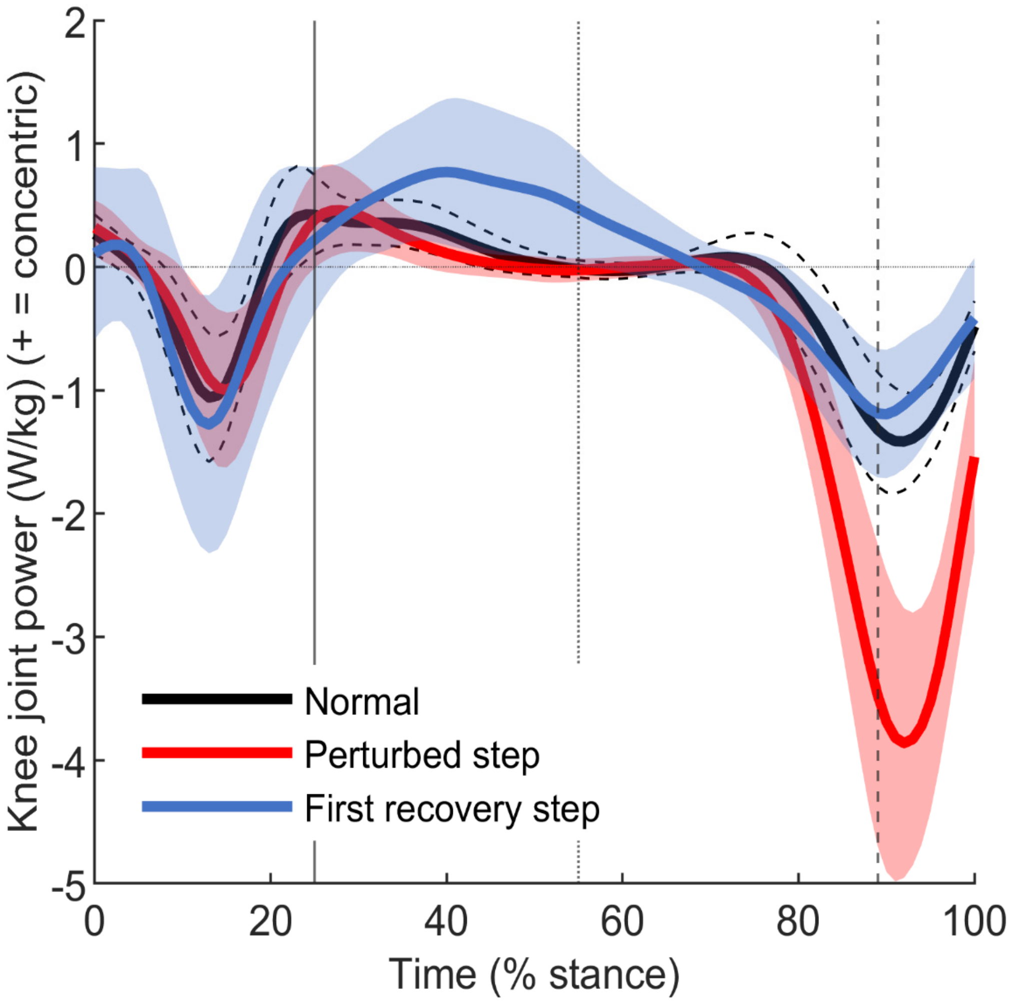

Figure S1. Knee joint power over the stance phase collected during normal gait (average of all participants thick black line ± 1SD dashed lines), the perturbed step (average of all participants thick red line ± 1SD shaded area) and the first recovery step (average of all participants thick blue line ± 1SD shaded area). The vertical black line at 25% stance represents the beginning of the belt acceleration, the dotted vertical black line at 55% stance represents the time of peak belt speed, the dashed vertical black line at 89% stance represents the time of peak knee moment during propulsion of the perturbed stance.

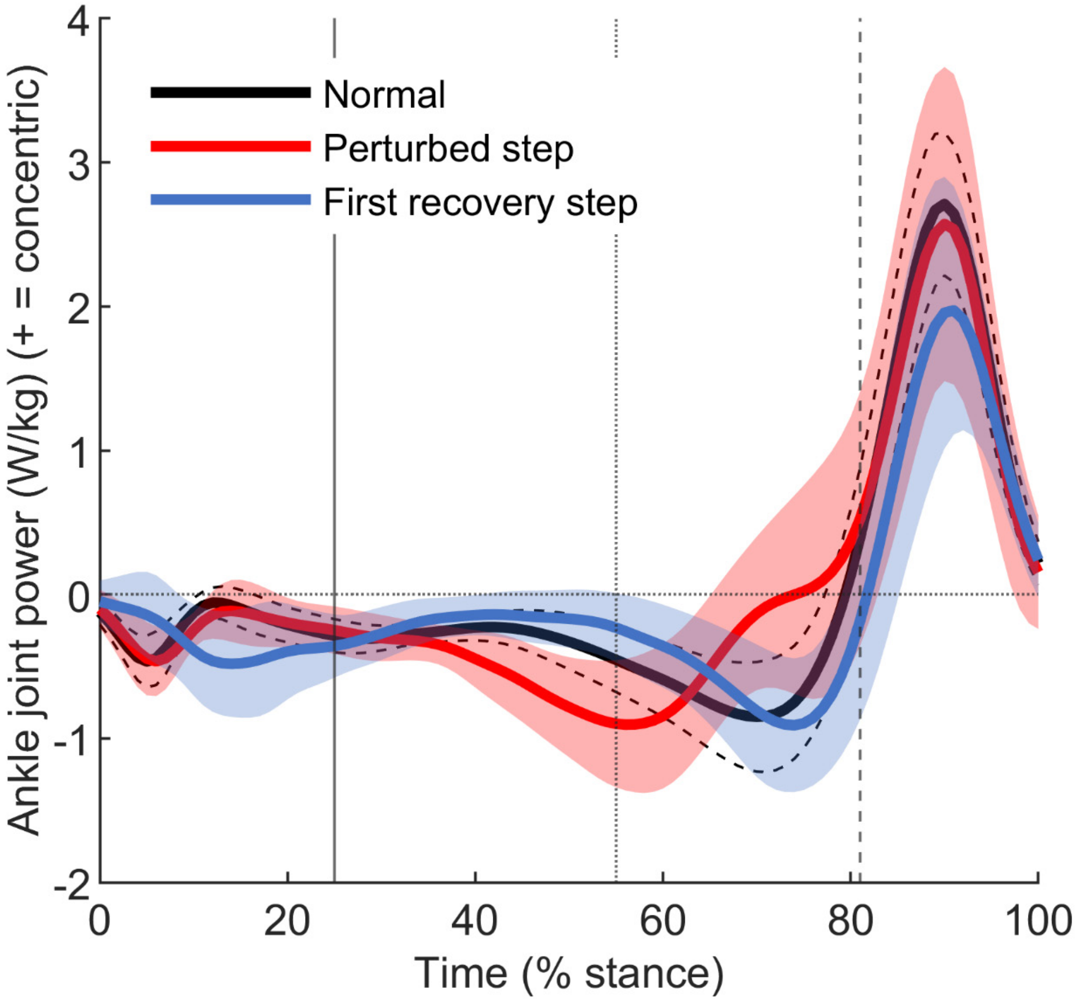

Figure S2. Ankle joint power over the stance phase collected during normal gait (average of all participants thick black line ± 1SD dashed lines), the perturbed step (average of all participants thick red line ± 1SD shaded area) and the first recovery step (average of all participants thick blue line ± 1SD shaded area). The vertical black line at 25% stance represents the beginning of the belt acceleration, the dotted vertical black line at 55% stance represents the time of peak belt speed, the dashed vertical black line at 81% stance represents the time of peak ankle moment during propulsion of the perturbed stance.

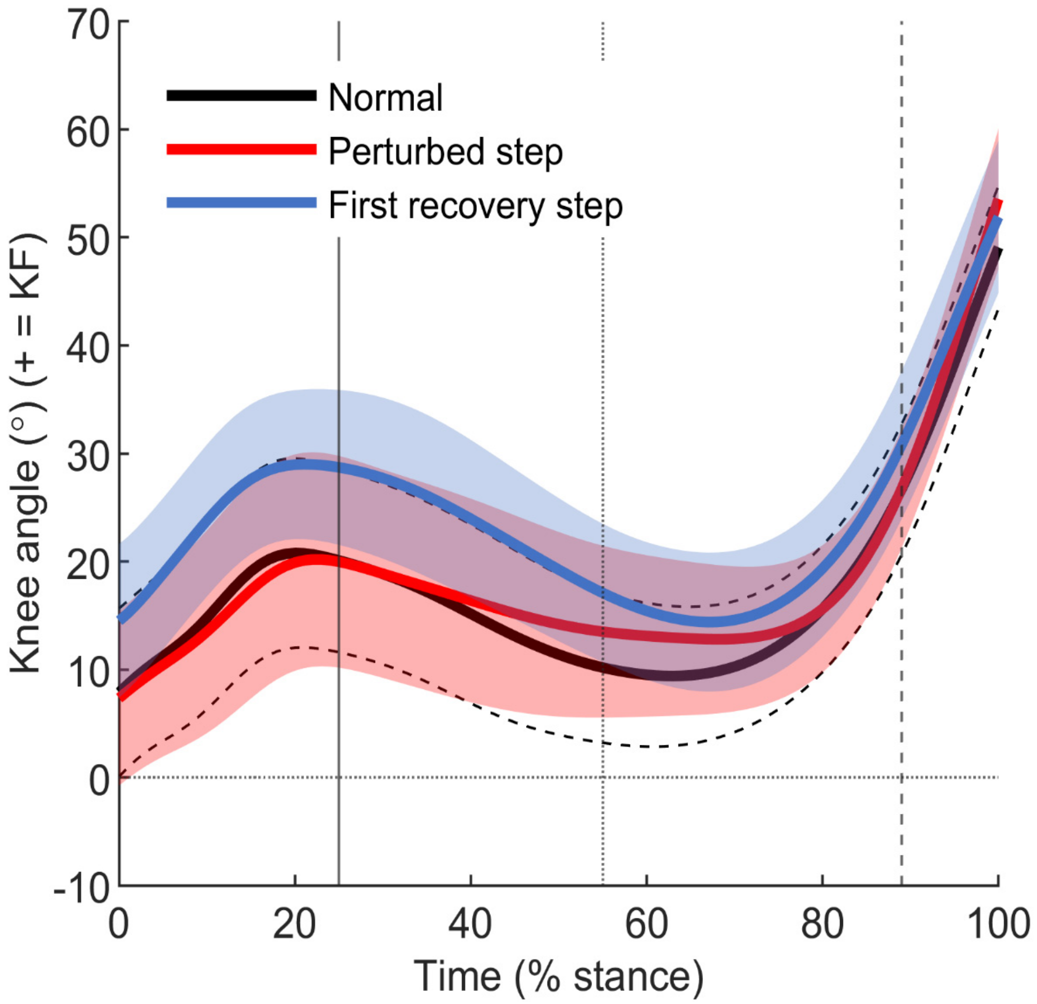

Figure S3. Knee angle over the stance phase collected during normal gait (average of all participants thick black line ± 1SD dashed lines), the perturbed step (average of all participants thick red line ± 1SD shaded area) and the first recovery step (average of all participants thick blue line ± 1SD shaded area). The vertical black line at 25% stance represents the beginning of the belt acceleration, the dotted vertical black line at 55% stance represents the time of peak belt speed, the dashed vertical black line at 89% stance represents the time of peak knee moment during propulsion of the perturbed stance.

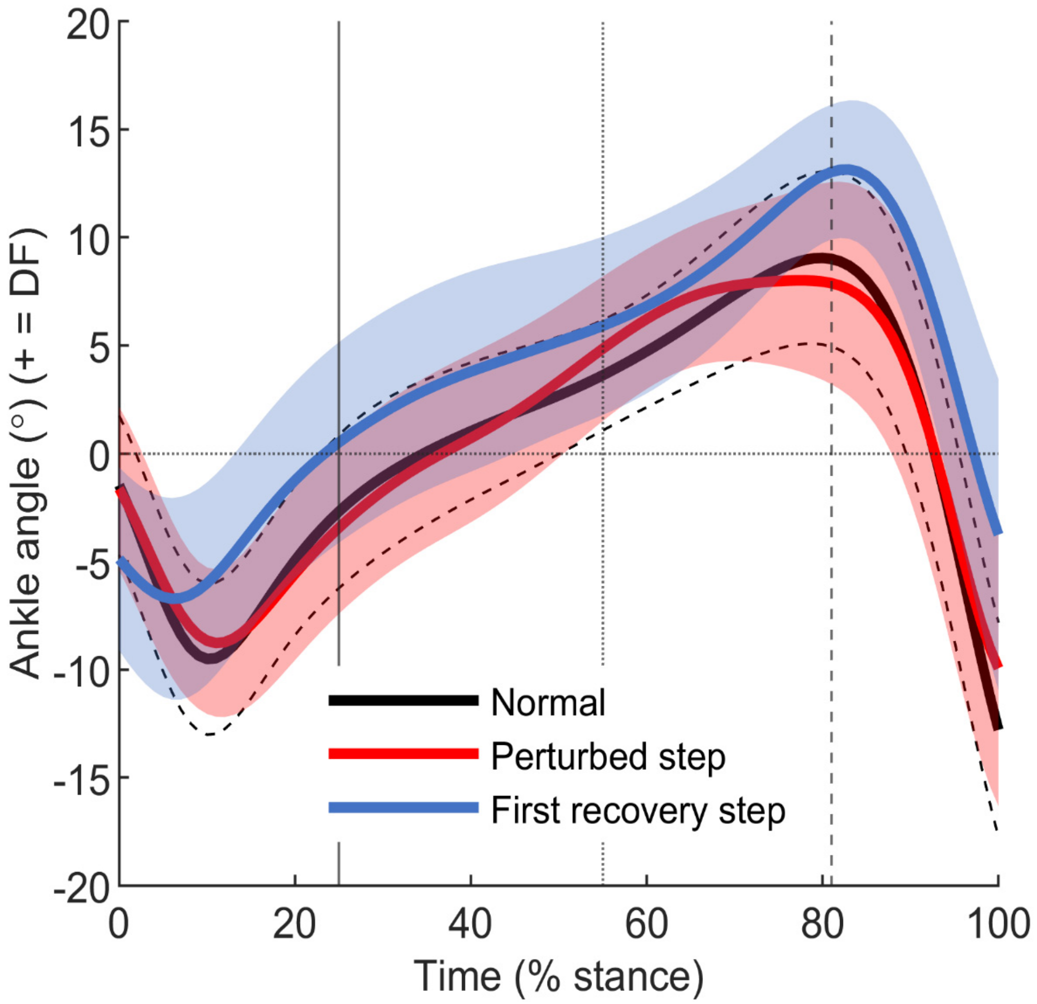

Figure S4. Ankle angle over the stance phase collected during normal gait (average of all participants thick black line ± 1SD dashed lines), the perturbed step (average of all participants thick red line ± 1SD shaded area) and the first recovery step (average of all participants thick blue line ± 1SD shaded area). The vertical black line at 25% stance represents the beginning of the belt acceleration, the dotted vertical black line at 55% stance represents the time of peak belt speed, the dashed vertical black line at 81% stance represents the time of peak ankle moment during propulsion of the perturbed stance.

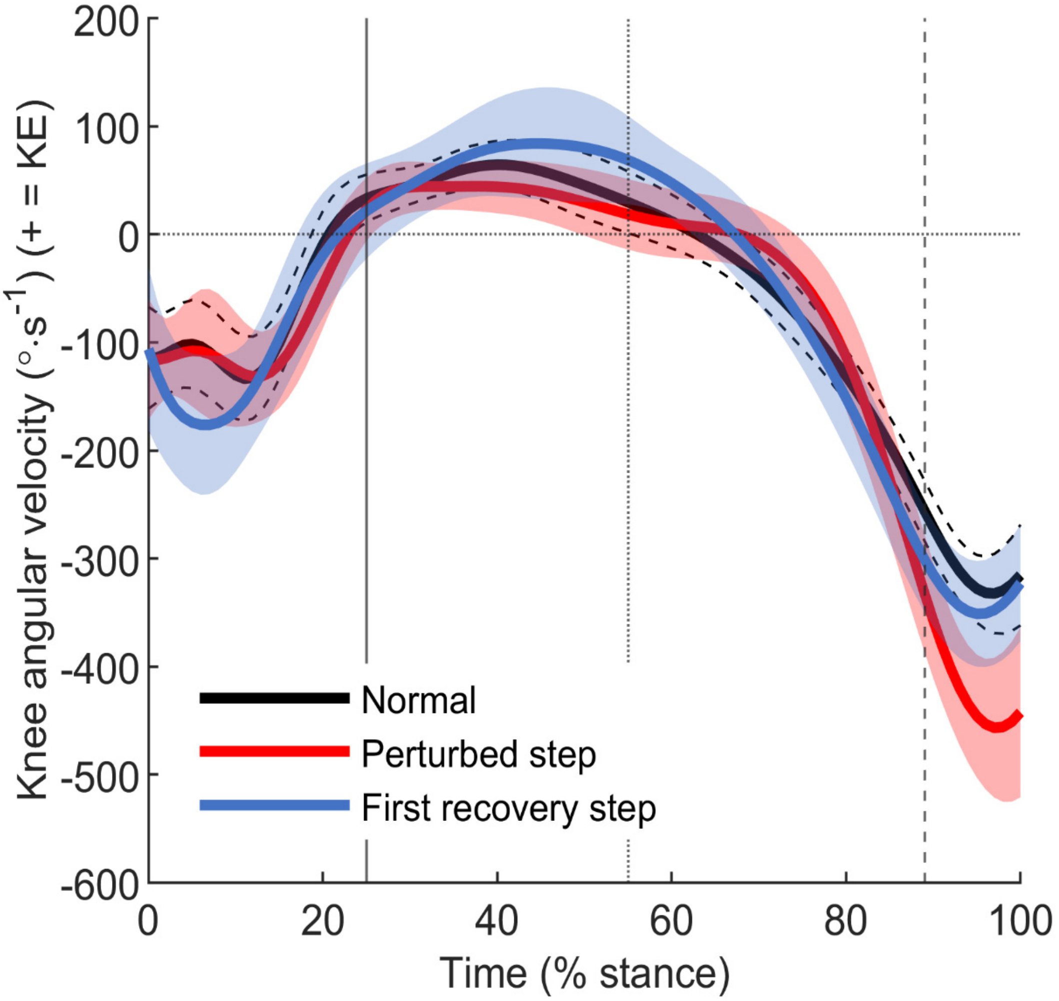

Figure S5. Knee angular velocity over the stance phase collected during normal gait (average of all participants thick black line ± 1SD dashed lines), the perturbed step (average of all participants thick red line ± 1SD shaded area) and the first recovery step (average of all participants thick blue line ± 1SD shaded area). The vertical black line at 25% stance represents the beginning of the belt acceleration, the dotted vertical black line at 55% stance represents the time of peak belt speed, the dashed vertical black line at 89% stance represents the time of peak knee moment during propulsion of the perturbed stance.

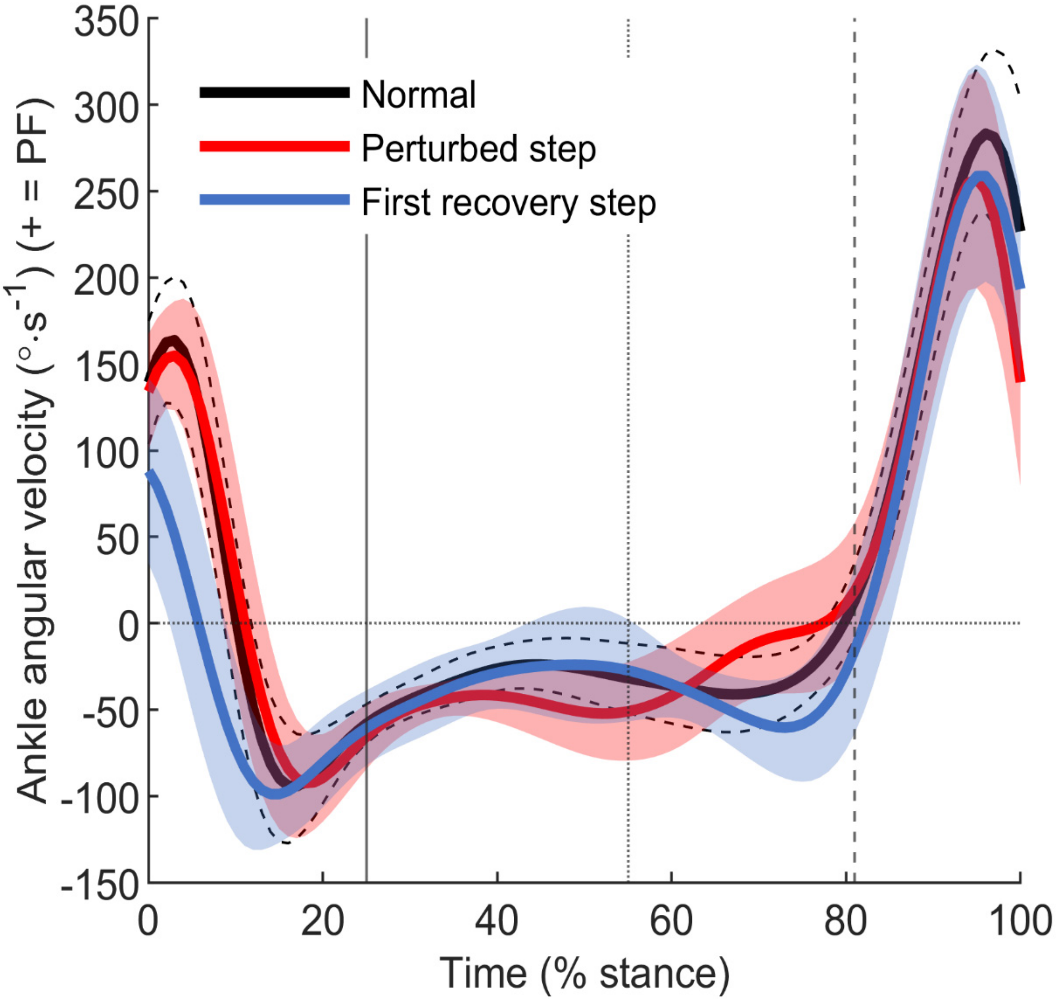

Figure S6. Ankle angular velocity over the stance phase collected during normal gait (average of all participants thick black line ± 1SD dashed lines), the perturbed step (average of all participants thick red line ± 1SD shaded area) and the first recovery step (average of all participants thick blue line ± 1SD shaded area). The vertical black line at 25% stance represents the beginning of the belt acceleration, the dotted vertical black line at 55% stance represents the time of peak belt speed, the dashed vertical black line at 81% stance represents the time of peak ankle moment during propulsion of the perturbed stance.
